# miRNA-Mediated Mechanisms in the Generation of Effective and Safe Oncolytic Viruses

**DOI:** 10.3390/pharmaceutics16080986

**Published:** 2024-07-25

**Authors:** Mariia Toropko, Sergey Chuvpilo, Alexander Karabelsky

**Affiliations:** Gene Therapy Department, Sirius University of Science and Technology, Olympic Avenue, 1, 354340 Sochi, Russia; chuvpilo.sa@talantiuspeh.ru (S.C.); karabelskiy.av@talantiuspeh.ru (A.K.)

**Keywords:** oncolytic virus, virotherapy, recombinant viruses, microRNA, oncological diseases

## Abstract

MicroRNAs (miRNAs) are short non-coding RNAs that regulate gene expression by inhibiting the translation of target transcripts. The expression profiles of miRNAs vary in different tissues and change with the development of diseases, including cancer. This feature has begun to be used for the modification of oncolytic viruses (OVs) in order to increase their selectivity and efficacy. OVs represent a relatively new class of anticancer drugs; they are designed to replicate in cancer tumors and destroy them. These can be natural viruses that can replicate within cancer tumor cells, or recombinant viruses created in laboratories. There are some concerns regarding OVs’ toxicity, due to their ability to partially replicate in healthy tissues. In addition, lytic and immunological responses upon OV therapy are not always sufficient, so various OV editing methods are used. This review discusses the latest results of preclinical and clinical studies of OVs, modifications of which are associated with the miRNA-mediated mechanism of gene silencing.

## 1. Introduction

Cancer is one of the leading causes of death in the world [1]. The International Agency for Research on Cancer (IARC) registered about 20 million new cases of the disease in 2022, of which 9.7 million were lethal [2], and this number is predicted to increase [1]. Along with the use of traditional treatment methods (chemotherapy, radiotherapy, and the surgical removal of tumors), in recent years, targeted therapy methods for oncological diseases have been actively developed: monoclonal antibodies, small molecule inhibitors of signaling pathways, antibodies conjugated with cytotoxic agents, stem cells, nanoparticles of various natures, viruses, etc. [3,4]. Oncolytic viruses (OVs) selectively replicate in cancer cells and destroy them without affecting healthy tissues. OVs act by directly killing cancer cells as well as by activating the immune response against tumors. Two types of OVs can be distinguished: (1) viruses that are not pathogenic to humans and capable of natural multiplication primarily in cancer cells, and (2) OVs created using genetic engineering [5]. Recombinant OVs are a relatively new class of anticancer drugs that have demonstrated therapeutic efficacy in the treatment of melanoma [6], squamous cell carcinoma of head and neck (SCCHN) [7,8], bladder cancer [9], brain cancer [10], colorectal cancer [11], non-small cell lung cancer [12], and other cancer types, as well as in combined therapy [13,14].

In medical practice, there are many examples of partial, temporary, or complete cancer remission after a viral infection or vaccination [15,16,17,18,19]. However, natural viruses as oncolytic viruses can cause undesirable side effects, due to their ability to infect healthy tissues. Moreover, their oncolytic properties can be insufficient, and they can be destroyed by the patient’s pre-existing immunity [16,19]. Therefore, based on natural viruses, oncolytic recombinant viruses are designed with additional modifications that increase their efficiency and specificity. Among OV modifications used in practice are the insertion of genes encoding immunostimulating or antiangiogenic factors [20,21,22], pseudotyping [23], and mutagenesis [24,25]. To reduce the neutralization of OVs by the immune system, they are coated with nanomaterials [26,27] or delivered in carrier cells obtained from a patient [28]. The increased tumor selectivity of OVs can be achieved via mutations in genes associated with virulence or genome replication [29,30] or by deleting certain genomic sequences [31], as well as by placing genes under the control of tumor-specific promoters [32,33,34,35]. The surface proteins of OVs can be modified with antibody fragments or ligands for certain receptors presented on target cancer cells [36]. Also, to increase tumor specificity, genes responsible for bypassing antiviral cell defense mechanisms are removed from OVs [29,37,38]. OVs themselves can carry interferon genes, which activate the antiviral response in healthy cells [39,40,41,42,43].

Regulatory non-coding RNAs, which control gene expression at the post-transcriptional level, are also used for OV modification. microRNAs (miRNAs) represent non-coding RNAs that regulate many important processes in different organisms. The relative abundance of miRNAs in cells can differ during tumorigenesis, and some miRNAs act as tumor stimulators or tumor suppressors [44]. These features are used for cancer therapy with OVs.

This review aims to present the current state of research on miRNA-modified OVs, discuss the results of the first clinical trials of miRNA-controlled OVs, and outline future developments in this field.

## 2. Control of Gene Expression via miRNAs

miRNAs are non-coding RNAs of 18–22 nucleotides in length. Being a component of the RNA-induced silencing complex (RISC), they modulate gene expression by degrading or repressing the translation of target mRNAs, although it has recently been shown that some miRNAs, such as miR-212-5p and miR-221-5p, increase the translation of target transcripts through direct interaction with them [45]. miRNAs are involved in the regulation of embryogenesis, organogenesis, stem cell proliferation, apoptosis, aging processes, hematopoiesis, and cancer development [46,47].

miRNAs can originate from intronic, exonic regions of the genome, as well as from intergenic regions [48]. In the canonical pathway, the precursor miRNA, the so-called primary miRNA (pri-miRNA), is transcribed by RNA polymerase II in the nucleus and folded into a hairpin. Then, type III RNase Drosha, with the help of two auxiliary molecules of DGCR8 (Pasha in fruit flies and nematodes), cuts each of two strands in the hairpin with an offset of two nucleotides, resulting in the formation of a pre-miRNA with a shortened hairpin structure [49]. Following transport from the nucleus by exportin V (XPO5) and Ran-GTP into the cytoplasm, the pre-miRNA is cut by RNase III Dicer near the loop, forming a double-stranded RNA molecule that consists of the guide and its complementary passenger strand (miRNA*) [50]. The resulting RNA duplex is loaded into the Argonaute protein (Ago) with the help of the HSC70/HSP90 chaperone proteins [51]. Here, the two RNA strands are separated, and one of them becomes a mature miRNA, while the other is removed. Each of the RNA duplex strands can be selected as a guide RNA in the RISC. Usually the one whose 5′ end is less stably bound to the complementary strand is selected [52,53].

In a miRNA, a “seed” sequence, which usually includes nucleotides from the 2nd to the 8th, complementarily binds to the target sequence in mRNAs. In addition to the “seed” sequence, more distal nucleotides are also involved in specific miRNA binding [49]. miRNA response elements (MREs) in mRNAs are usually localized in 3′-untranslated regions (3′-UTRs) in Metazoans, as well as in 5′UTRs and in coding regions. Depending on the complementarity degree between miRNAs and their MREs, several mechanisms of transcript inhibition are possible. The main inhibition mechanism is the recruitment of additional proteins into the RISC, which leads to transcript degradation [54,55]. In this case, the mammalian adapter protein TNRC6 (homolog of GW182) of the RISC interacts with the poly(A)-binding protein, which recruits PAN2–PAN3 and CCR4–NOT deadenylase complexes [56]. The poly(A)-tail shortening leads to transcript destabilization and translation inhibition. Then, the cap is removed by DCP1/2, and the 5′- and 3′- ends are degraded by exonucleases [57,58]. Rarely, in mammals, mRNAs are endonucleolytically cleaved by the Ago2 protein, in the case of significant complementarity of the seed sequence and MRE [59,60].

miRNAs are fairly stable molecules, possibly due to their short length and inclusion in protein complexes. In the body, miRNAs are secreted by cells on their own or enclosed in membrane vesicles—exosomes or microvesicles. This is a unique communication method between cells, which is also used by cancer tumors to influence their environment’s homeostasis. Currently, drugs based on miRNA mimics, that is, artificially synthesized double-stranded RNAs, including small interfering RNA (siRNA) sequences and primary microRNA transcripts (pri-miRNAs), are undergoing clinical trials [61,62]. Despite the relative stability of miRNAs, there are limitations for their systemic administration, such as a susceptibility to nuclease degradation and the rapid clearance of miRNAs from the blood, immunotoxicity, their inert and nonspecific uptake into cells, and the lack of control of the miRNA dose per cell [62]. These problems are partially solved by the use of various methods of delivering miRNAs to tissues. These are non-viral, such as liposomes, polymers, metal nanoparticles, extracellular vesicles, and bacterial non-living mini-cells, as well as conjugation with aptamers and other molecules, and viral methods [61,63]. Recombinant viral vectors stand out with their quite accurate delivery of genetic material to target cells and their ability to ensure transgene stability.

There is increasing evidence that miRNAs play a significant role in virus–host cell interactions [64]. Many viruses encode their own miRNAs, which regulate either the life cycle of a virus itself [65,66,67], or the expression of host cell genes responsible for cell cycle control, activation of the immune response, regulation of apoptosis, etc. [68,69,70,71,72,73]. This feature was taken into account by Otani Y. et al. for oncolytic HSV-1 glioma therapy [74]. The HSV-1-encoded miR-H16 targeting factor FIH-1 which inhibits HIF-1, was used. This activated NOTCH signaling, which is often required by glioma cells for survival. Researchers combined the action of the OV with a NOTCH-blocking gamma secretase inhibitor to improve the effectiveness of the therapy.

Most often, viruses containing double-stranded DNA encode their own miRNAs [75]; however, RNA viruses are also capable of synthesizing miRNAs [76]. It was previously shown that recombinant RNA viruses can ensure normal miRNA production while maintaining their replicative activity and genome integrity [77].

Viral miRNA precursor transcripts are processed by the same enzymes as cellular miRNAs [64]. In the case of viruses which replicate in the cytoplasm, their miRNA precursors are processed by the enzyme Drosha, which, under the influence of a viral infection, is translocated from nucleus to cytoplasm [76,78,79], but this translocation does not always happen [80].

Some mammalian miRNAs suppress [81,82,83] or, conversely, promote virus replication [84]. For example, miR-99b and miR-485 promoted oncolytic adenovirus replication in pancreatic adenocarcinoma cells [85], miR-26b increased the production of adenoviruses in prostate cancer cells [86] and lung carcinoma [87], miR-31 and -128 reduced the infectivity of a measles virus by targeting poliovirus receptor 4 (PVRL4), which is the entry receptor for this virus [88], and miR-4521 inhibited Newcastle disease virus (NDV) replication in HeLa cells [89]. Also, upon viral infection, the miRNA expression level can change in the host cell, e.g., infection with adenovirus of serotype five led to a decrease in miRNA level in prostate cancer cells [86] and NDV affected the expression of several miRNAs in HeLa cells [89].

## 3. Diversity of miRNAs Used to Modify Oncolytic Viruses

Each gene is regulated by a number of miRNAs, and each miRNA regulates thousands of targets, so one miRNA can control several intracellular signaling pathways. Therefore, targeting even one miRNA can lead to significant changes in cell metabolism.

**Figure 1:**
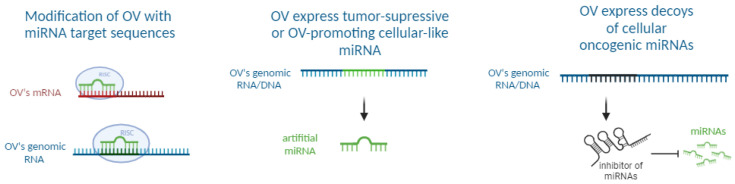
Current oncolytic virus modification strategies associated with microRNA-mediated gene silencing mechanisms. This figure was created with BioRender.com (https://biorender.com/; Accessed on 21 July 2024).

The choice of a miRNA for OV modification is based on the targeted cancer type, virus modification strategy, and miRNAs’ differential expression levels in a given cancer type as well as in normal tissues. To date, three strategies for the modification of OVs are known, based on the mechanism of gene suppression by miRNA (Figure 1), as follows:

(1) **miRNA target site insertion:** This strategy involves integrating miRNA target sites (miRTs) into viral genomes. miRNAs can bind to miRTs and inhibit specific mRNA translation, thereby restricting viral replication primarily within healthy cells. This is the most common approach.

(2) **Artificial miRNA expression**: this approach entails introducing synthetic miRNA expression cassettes to inhibit proto-oncogenes expression and/or to enhance OV replication within cancer cells.

(3) **miRNA sponge**: This strategy involves inserting artificial or naturally occurring RNA sequences into the viral genome. These sequences might contain multiple binding sites specifically designed to capture and inactivate some cellular encoded miRNAs, effectively reducing their activity within the cell.

miRNAs chosen to modify OVs in the last 10 years are listed in Table S1 (Supplementary Materials, Table S1). In these studies, nervous system-specific miRNAs were most often selected, such as miR-124, -128, -125, and -7, since it was necessary to limit the replication of OVs in healthy nerve tissues. In these studies, OVs were targeted not only to cancers of neuronal origin, but to a variety of cancer types, and such modification was typically required for viruses that have a natural tropism for neural tissues. miR-124 was most often chosen, since this miRNA is abundant in the central nervous system of mammals, and its level is reduced in many cancers of neuronal origin [90,91,92].

miRTs are introduced into the genes important for viral replication, which ensures the specific expression of an OV. To date, this method is used more often compared to the remaining two—the viral synthesis of artificial miRNAs or of miRNA inhibitor molecules. Typically, to regulate an OV’s gene expression, miRNAs are selected, the level of which is reduced in cancer cells but increased in normal tissues.

In the case of artificial miRNA synthesis by OVs, it is reasonable to choose the so-called tumor-suppressive miRNAs, which regulate the expression of cancer-associated genes, and the level of which often decreases during oncogenesis. In recent studies, miR-143 was most often chosen when creating OVs encoding miRNAs. This miRNA is considered to have a tumor inhibitory effect and has an elevated level in smooth muscle cells and a decreased one in many types of cancer [93]. Researchers also selected miRNAs largely presented in the nervous system (miR-99b, -485, and -34a) [94,95,96], skeletal and smooth musculature (miR-1) [97,98], in liver (miR-122) [98,99], lungs (miR-34c) [96,100], or ubiquitous (miR -34a and let-7) [96,101].

It should be noted that the same miRNA can act as both a tumor suppressor and an oncogenic miRNA [102]. For example, miR-125b plays an oncogenic role in some cancer types like colon cancer and hematopoietic tumors, while in others it is tumor-suppressive [103,104]. Other examples include miR-216, -142, -20a, 10a/b, -151, -155, 181a/b, -184, -501, -9, -29a, -373, -199a, -217, and -204 (Supplementary Materials, Table S1).

OVs may also carry inhibitors of oncogenic miRNAs that suppress the expression of genes important for preventing tumorigenesis. Currently, there are few reports devoted to the creation of such OVs. Most of them inhibit a large number of miRNAs simultaneously, which enhances the viral anticancer effect.

## 4. Preclinical Studies of Oncolytic Viruses Modified with miRNA Recognition Sites

Recombinant OVs have been shown to be effective against cancer in clinical trials, especially when combined with chemotherapy or immune checkpoint inhibitors [105]. However, there still remains a problem of insufficient specificity of OVs to cancer cells and, as a consequence, side effects and toxicity, which are enhanced during combinatorial therapy [105,106].

**Figure 2:**
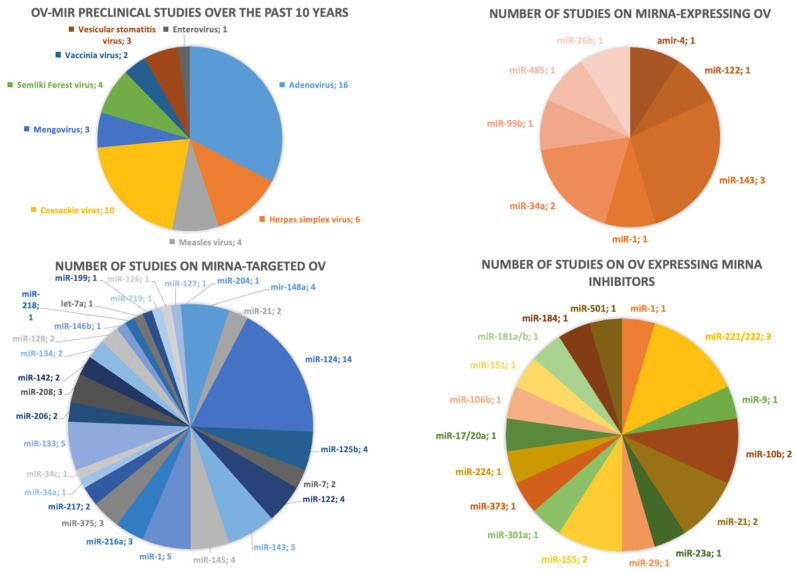
Analysis of oncolytic viruses reviewed in this article. The data were visualized in Microsoft Excel 2016.

One strategy for increasing oncoselectivity is to introduce into an OV’s genome target sequences for tissue-specific miRNAs, the expression of which are reduced in tumors (Figure 2). Previous studies have shown that this approach effectively controls the propagation of OVs. miRTs attenuate the level of virus in normal cells and thus reduce its toxicity when administered systemically.

### 4.1. Application of miRTs for Oncolytic Viruses Detargeting

It has been shown that increasing the number of miRTs can increase OVs’ oncoselectivity [107,108], but not always [109]. The miRTs’ position is important, and targeting multiple genes appears to be more effective [110]. The combination of target sites for several miRNAs simultaneously makes an OV safer and limits an OV’s replication in different tissues and organs [111,112], which could potentially be at risk when administered systemically [113]. Targeting viral early phase proteins turns out to be more effective, since, in this way, a decrease in the late phase protein level is also achieved [114].

miRNA selection to target an OV depends on cancer type, the backbone virus, and the organs which should be protected. In recent research, certain miRNAs have been more often selected. These are miR-124-3p, which is abundant in the nervous system, miR-133, which is increased in skeletal and cardiac muscle tissues, miR-143, which is enriched in smooth muscles of blood vessels and internal organs, and miR-1, the expression of which is elevated in smooth, skeletal, and cardiac muscles (Supplementary Materials, Table S1).

miRNA target sites can be placed in different sites of an OV’s genome. In (-)RNA viruses, a gradient of gene expression depends on the position of the particular gene relative to the 3′ end of the genomic RNA [115]. In oncolytic vesicular stomatitis virus (VSV), targeting the distally located L gene, which is expressed in the smallest amount and encodes the viral RNA polymerase, was more effective than targeting the matrix protein M gene [116]. However, in the measles virus, the introduction of miRTs in proximally located genes turned out to be more effective than in distal ones [109].

In several studies, different positions of miRTs in the (+)RNA-genome of oncolytic coxsackieviruses (CVs) had different effects. In the report of Jia Y. et al. [117], oncolytic CVB3 with miRTs in the 3′-UTR turned out to be safer in biochemical tests than a virus with miRTs in the 5′-UTR. At the same time, a virus with double inhibition, with targets for miR-34a in both the 5′-UTR and 3′-UTR of genomic RNA, was the least toxic and demonstrated a good oncolytic effect on lung cancer tumors in xenograft models. In other studies, the oncoselectivity of CVB3 was successfully increased by introducing miRTs into the 3′-UTR of the genome [118,119,120] or into the 5′-UTR [121,122,123,124]. For the CVA21 virus, the most tolerant was the introduction of transgenes closer to the 3′ end of the 5′-UTR of the genomic RNA [125,126]. For another Picornaviridae member, mengovirus, the effect of the introduction of miRTs both in the 5′- and 3′-UTR was higher than in each site separately [127].

Depending on the type of viral genome, it is advantageous to target either the viral mRNA or its genome. In DNA viruses, miRTs are introduced in such a way that sequences complementary to the miRNA are localized in mRNAs. Both mRNA and genomic RNA are targeted in (+)RNA viruses, which seems to be an effective method for increasing tumor selectivity. However, for (-)RNA viruses, such double control is impossible due to rapid genome encapsulation, which occurs almost simultaneously with replication and transcription [128]. Attempts at such modification were made in influenza virus and VSV, but they were unsuccessful [116,129]. For this reason, miRTs in (-)RNA viruses are introduced in such a way that miRNAs interact with mRNAs of genes crucial for viral replication.

### 4.2. Issues Regarding miRT Usage and Their Solutions

Due to the reduced accuracy of RNA polymerases, the probability of mutations in RNA viruses is higher than in DNA viruses, so nucleotide substitutions may appear in miRTs of recombinant OVs with an RNA genome. This can lead to the impaired binding of miRNAs to target sites, decreased control over OV replication, and increased toxicity [127]. The combination of recognition sites for several miRNAs, the synthesis of which partially coincides in different tissues, as well as an increase in miRT numbers can help reduce the likelihood of viruses escaping from the control of miRNAs. The normal functioning of the patient’s immune system is also necessary.

It is possible that after several generations, viruses can lose their miRTs. The solution may be to stabilize a virus’ genome. For example, deletion of the polycytidine tract in mengovirus vMC24-NC led to genome stabilization and the preservation of miRTs [130]. The virus was effective against plasmacytoma tumors in vivo.

There are many examples of successful targeting of RNA viruses with miRTs. Recombinant coxsackievirus CVB3 retained the integrity of miRTs after four serial passages in cell lines in vitro [121], CVA21 succeeded after seven passages [125], and mengovirus vMC24-NC succeeded after ten passages [127]. In addition, mutations and losses of miRTs did not always lead to toxic effects [127]. It is possible that normally functioning miRTs can lead to OV inactivation in normal tissues before mutations occur. Unfortunately, researchers do not always assess the stability of miRTs in OVs. It is clear that further study of miRTs’ integrity in rapidly evolving RNA viruses is required.

### 4.3. Adenoviridae

Viruses controlled by miRNAs are presented in Table 1. Oncolytic adenoviruses are among the most commonly modified with miRTs, and of all serotypes, serotype 5 is most often chosen [86]. Adenoviruses are non-enveloped viruses with a double-stranded linear DNA genome of ~36 kb and with an icosahedral capsid [131]. These viruses have high genome stability, relatively high capacity, and a broad tropism for tissues of various origins, which allows their usage against cancers of numerous etiologies.

Limiting the propagation of oncolytic adenovirus using the RNA interference mechanism reduced its accumulation in the liver and made it less toxic when administered systemically [114]. Eight sequences fully complementary to miR-148a, which is abundant in the pancreas and liver, were introduced into the 3′-UTR of the L5 gene, which encodes the fiber protein [151,152,153]. OV’s oncolytic activity was preserved, and it induced an antitumor response in mice with patient’s tumors.

In the report of Yuan et al. [32], the *E1A* gene of oncolytic adenovirus was successfully placed under the control of the alpha-fetoprotein promoter and miR-122, which is abundant in adult liver cells. To more accurately deliver the virus to hepatocellular carcinoma tumors, human umbilical cord mesenchymal stem cells (MSCs) were used. MSCs were transported through the bloodstream to the liver, differentiated into hepatocyte-like cells within the tumor microenvironment, and produced oncolytic adenovirus. The resulting drug was planned to be administered intravenously and was developed to combat metastases and tumor remnants after surgical removal.

In one study, to increase the tumor specificity of an oncolytic adenovirus and control the expression of immunomodulatory molecules, a genetic circuit was developed. Viral genes were placed under the control of a tumor-specific promoter and miRTs specific for normal or tumor tissues [34]. A method for the circuit’s assembly (about 6.5 kb) and cloning it into an adenoviral core vector was also presented. An oncolytic adenovirus against hepatocellular carcinoma (HCC) was created using such a circuit, which included the activator Gal4VP16, two mutually inhibiting repressors LacI and tetR:Krab, a tumor-specific α-fetoprotein promoter, and sequences for binding tumor-specific miR-21 and normal hepatocyte-specific miR-199- 3p and miRNA-142. Viral replication was high when the production of miR-21 was increased and the production of miR-199-3p and miR-142 was decreased. Several virus variants also expressed immunomodulatory molecules: IL-2, murine GM-CSF, anti-PD-1-scFv, or anti-PD-L1-scFv. These OVs limited the growth and reduced the volume of tumors in xenograft murine models and led to the development of an antitumor immune response. In addition, the authors, based on the computer model they developed, showed that sequential administration of an agent and an immunomodulatory agent is therapeutically more effective than their simultaneous use. This effect can be achieved by using a virus encoding immunomodulatory molecules in therapy. The authors note that it is necessary to study the dynamics of immunomodulators delivered by the virus and determine their safe dose.

The expression of transgenes delivered by OVs can also be controlled using miRNAs. Several variants of an oncolytic adenovirus encoded single-stranded membrane-bound IL-12, which was controlled by miR-122 targets [133]. Such control of cytokine expression limited its distribution into the bloodstream and ensured its local and, as a consequence, more promising effect with a lower dose of OV and reduced toxicity of the drug.

### 4.4. Herpesviridae

HSV types 1 and 2 are also both used as the basis for the creation of OVs. HSV belongs to the Herpesviridae family; these are large enveloped viruses with an icosahedral capsid and a large genome (152 kb), presented in the form of double-stranded DNA [154]. Large transgenes can be cloned into them; about 30 kb in the HSV genome can be deleted or replaced with other sequences without a critical decrease in its ability to replicate [155]. The first OV approved by the Food and Drug Administration (FDA) in 2015 for the treatment of melanoma was recombinant HSV-1 (T-Vec, Imlygic™), encoding granulocyte–macrophage colony-stimulating factor (GM-CSF) to promote immune response against tumors [156]. HSV’s natural ability to infect neurons makes it an excellent platform for targeting neuronal cancers, but HSV has also been used to target other forms of cancer. The downside of neural tissue tropism is that HSV is potentially neurotoxic and can establish a latent infection of neurons. Introducing miRTs has proven to be an effective way to improve the safety of HSV-based oncolytic viruses.

Oncolytic HSV-1 against glioblastoma was studied in the work of Mazzacurati L. et al. [136]. Sequences complementary to miR-124, abundant in neurons but decreased in glioblastoma cells, were introduced into the 3′UTR of the important ICP4 gene. The virus replicated in spheroids from two glioblastoma lines, Gli68 and GBM30, at rates comparable to similar viruses without the miRTs. The OV also increased the survival of mice engrafted with aggressive patient-derived GBM30 tumors. When administered intracranially to immunodeficient mice, the virus caused no pathological changes during the 33-day observation period. Additionally, miRTs did not affect the oncolytic efficacy of an OV variant targeting EGFR/EGFRvIII receptors, which are highly expressed on cancer cells.

The control of OVs to reduce toxicity can be carried out at the transcriptional, post-transcriptional, and translational levels simultaneously, as demonstrated by Delwar Z. et al. [33]. The ICP4 gene of an oncolytic HSV-1 was placed under the tumor-specific survivin promoter, and miRTs were introduced into the 3′UTR of the ICP4 gene. Additionally, the 5′UTR of the rat fibroblast growth factor 2 (*FGF2*) gene was placed upstream of the ICP4 open reading frame. FGF2 requires high levels of eukaryotic translation initiation factor 4 (eIF4E) for successful translation, and its expression is increased in glioma cells but not in normal neurons [33]. This approach has proven to be effective against glioma, and it reduces OV’s neurotoxicity in vitro and in vivo.

An unusual example in the literature shows that the production of oncolytic herpes simplex virus (HSV) increased in the presence of oncogenic miR-21, which is elevated in cancer cells [135]. The UL9 protein is necessary for HSV to initiate replication. The HSV-based OV expressed dnUL9 peptide that inhibits binding of UL9 to the viral origin of replication. Due to miRTs in the dnUL9 sequence, dnUL9 expression was suppressed in the presence of miR-21, and the virus replicated.

### 4.5. Poxviridae

Vaccinia virus (VV) is a large enveloped virus that replicates in the cytoplasm and so is non-integrative [157]. Its genome is double-stranded DNA of approximately 200 kb, which allows the insertion of large amounts of foreign DNA [158]. It has the potential to destroy cancer cells; e.g., myeloma is partially susceptible to this virus. In one study, the tumor specificity of VV was increased by removing the thymidine kinase gene and inserting let-7a miRTs into 3′UTR of the *B5R* gene, which is important for virus envelopment and release [37]. The virus inhibited the development of myeloma in severe combined immunodeficiency (SCID) mice and extended their lifespan. In this case, OV led to local and more moderate skin damage in mice, compared to the control wild-type virus and a virus without miRTs, and did not harm important internal organs.

### 4.6. Picornaviridae

CVs are a group of small, non-enveloped viruses belonging to the genus Enterovirus of the Picornaviridae family [159]. OVs are created on the basis of both group A and group B CVs [160,161,162]. CVs are human pathogens which cause serious diseases. They enter cells through the CAR (coxsackie and adenovirus receptor) and DAF (decay-accelerating factor) receptors, which is the reason they have a wide tropism in a variety of human tissues [163]. CVs are interesting for creating OVs for several reasons. They do not integrate into genomic DNA, so there is no risk of insertional mutagenesis. The CV genome is a positive-sense single-stranded RNA, like most oncolytic miRNA-targeted RNA viruses [164], since miRNA in this case can target both mRNA of an important gene and genomic RNA.

Several viruses can be delivered to the body in the form of a transcript encoding the viral full-length genome, the so-called infectious RNA (iRNA) [165]. In cells, fully functional viruses are rescued with high efficiency from iRNAs [166]. This approach reduces viral immunogenicity and prevents neutralization by antibodies upon repeated administration. In addition, the iRNA does not need specific receptors to enter the cell, which allows it to destroy a variety of cancer cells. iRNA can be modified with miRTs for more specific targeting [125]. Skeletal muscle-specific miR-133 and miR-206 recognition sequences were introduced into the 3′- or 5′-non-coding regions (NCR) of the CV genomic RNA, which have specific secondary structures required for viral replication. In this study, all modifications located in the 5′-NCR controlled the OV more effectively than in the 3′-NCR. CV-iRNA, in which miRTs were cloned instead of nucleotides 631–698 (residues) in the spacer region in the 5′-NCR, led to the complete lysis of melanoma tumors in murine models, and toxic effects were either absent or manifested depending on the iRNA dose. With this modification, the stability of the virus genome was the greatest.

In another study, recombinant CVB3 with miRTs to miR-145, -143, -216, and -1 in the 5′-UTR was combined with the immunostimulating peptide melittin and a synthetic Toll-like receptor 9 agonist, CpG oligodeoxynucleotide, to combat breast cancer and melanoma [124]. The oncolytic and immunostimulating effects of these drugs acting synergistically were higher than their effects individually. Also, when used together, the survival rate of mice was higher, and there were no toxic effects.

The miRNA-based strategy was combined with directed viral evolution to increase the safety and effectiveness of the recombinant Coxsackie B3 virus PD-H strain against refractory cancer cells [132]. The OV-induced apoptosis in Colo320 cells was significantly stronger than the parental strain and caused no side effects in mice in vivo.

Enterovirus EV-71, also known as EV-A71, is another member of the Enterovirus genus with a single-stranded (+)RNA genome of 7400 nucleotides, which replicates in the host cell cytoplasm. This virus is a human pathogen, which causes hand-foot-and-mouth disease and neurological disorders [167]. In one study, EV-A71 was used for the first time as an OV against malignant glioma [140]. It induced the apoptosis of glioma cells and suppressed tumor growth in immunodeficient murine models. The addition of miR-124 recognition sites into the 5′-UTR of the virus genome reduced its neurotoxicity while maintaining oncolytic activity against glioma tumors injected subcutaneously and intracranially.

Mengovirus is a small non-enveloped virus, with a single-stranded (+)RNA genome. It has a wide tropism, infects many species of mammals and birds, and causes myocarditis, encephalitis, diabetes, and disorders of the nervous and reproductive systems [168]. In humans, mengovirus rarely causes serious illness, infection is often asymptomatic or accompanied by febrile illness [168], and it is known to have oncolytic activity. Previously, the toxicity of mengovirus was partially reduced by truncating the polycytidine tract in the genomic 5′-NCR, which led to a decrease in its virulence [31]. Ruiz A.J. et al. showed that the additional introduction of recognition sites of neurospecific and cardiac-specific miRNAs into the 5′- and 3′-NCR effectively reduces the toxicity of mengovirus when administered intratumorally and systemically to mice with plasmacytoma tumors [127]. At the same time, the resulting virus (vMC24-NC) delayed the growth of tumors and extended the lifespan of mice. In another study, vMC24-NC limited the development of U87-MG glioblastoma tumors in vivo, was generally less toxic when administered intracranially than a virus without miRNA recognition sites, and showed a complete absence of toxic effects when administered intratumorally [30]. At the same time, a similar virus without miRTs was more toxic, which affected the survival of murine models. Thus, the strategy of suppressing oncolytic mengovirus using miRNA was more effective than removing genes such as the leader gene.

### 4.7. Togaviridae

Semliki Forest virus (SFV) is an enveloped virus with a (+)RNA genome, a member of the Alphavirus genus of the Togaviridae family. Several strains of SFV are neurovirulent; they are able to penetrate the blood–brain barrier within the central nervous system (CNS) when administered systemically and replicate in neurons and oligodendrocytes [169]. Some strains can infect cells with a functioning interferon antiviral response, including several cancer cell lines. The neurovirulent strain SFV4 with miR-124 recognition sites was partially resistant to IFN-β and efficiently lysed astrocytoma and glioblastoma cells in vitro and in vivo, although some mice exhibited severe neurological symptoms, presumably due to OV’s escape of the miR-124 control [138].

The introduction of neuron-specific miRT-124, -125, and -134 into the 3′UTR of SFV4 led to a decrease in nerve cells’ infection and OV’s spread in the brains of mice [91]. The OV was effective in vivo without causing encephalitis and also activated an antiviral immune response. However, the virus lost the ability to kill cancer cells in a dose-dependent manner after they were preincubated with IFN-β or when the cells secreted their own IFN-β, suggesting that the OV was partially sensitive to the antiviral interferon response.

To increase the resistance of an OV to the type I IFN response and thus increase its oncolytic efficacy, researchers enhanced the virus from the previous study with the VV decoy receptor B18R, which binds IFN-β and reduces the activation of the interferon signaling pathway [137]. The virus destroyed glioblastoma cells in vitro and in vivo, but replicated in brain cells and was highly neurotoxic, apparently due to the loss of miRTs in some viruses. The insertion of the *B18R* gene could lead to a decrease in the stability of the virus genome, which could lead to the loss of miRTs. This is confirmed by the fact that a virus with miRT but without *B18R* did not cause neurotoxicity.

An additional four mutations in three genes of the SFV4 with target sites for miR-124 (SFV-AM6-124T) promoted viral replication [24]. The oncolysis of several glioma cell lines increased in the absence of the interferon response suppression by the virus. In an ex vivo brain model obtained from mice with GL261 gliomas and in an in vivo model, when administered intraperitoneally, the virus replicated in orthotopic glioma tumors and induced the apoptosis of cancer cells. When combined with anti-PD1 therapy, the virus stimulated an antitumor immune response.

### 4.8. Paramyxoviridae

The safety of measles virus-based OVs has also been improved by introducing miRTs. Measles virus is an enveloped virus with a single-stranded (-)RNA genome belonging to the genus Morbillivirus of the family Paramyxoviridae. It interacts through the H protein with the cellular receptors CD150 and nectin-4, which allows it to infect immune, epithelial, and endothelial cells, as well as with the CD46 receptor, expressed on all cells of the body except erythrocytes [170].

Oncolytic measles virus with increased tumor specificity, due to targets for miR-148a in the F gene, has been studied as a drug against pancreatic cancer [139]. Based on the most effective chemotherapy regimens for this type of cancer currently used in clinical practice, the effect of a combination of fluorinated pyrimidines with the OV was studied. The recombinant measles virus encoded a bacterial prodrug convertase, namely a hybrid protein of two enzymes—cytosine deaminase and uracil phosphoribosyltransferase—and, in combination with 5-fluorouracil, better suppressed tumor growth and prolonged the lifespan of mice with xenografts. In this case, not only OV-infected cells were destroyed, but also nearby uninfected cancer cells.

## 5. Preclinical Studies of Oncolytic Viruses Expressing miRNAs

As mentioned earlier, changes in miRNA levels occur during malignant transformation, including those involved in oncogenesis [44,171]. Regulating the expression of miRNAs in cells is a relatively new approach that is starting to be used in regenerative medicine, in the treatment of diabetes, viral infections, cancer, and other diseases [172,173,174,175]. Studies have been conducted on oncolytic viral vectors expressing oncosuppressive miRNAs, such as miR-34, -143, -99, -485, -1, -26b, and -122 (Table 1).

### 5.1. miRNA Precursors Delivered by Oncolytic Viruses

miRNAs can be delivered into cells in the form of different types of precursors. When a pri-miRNA-1 was delivered by an oncolytic adenovirus, the miR-1 expression level was the highest compared to the delivery as short hairpin RNA (shRNA), or pre-miRNA [87]. In other studies, the same approach was effective [141,142], or a cDNA encoding a miRNA was cloned into an OV [145] or artificial miRNAs [38,80]. Recently, the first oncolytic measles virus producing artificial miRNA was presented [80]. The OV encoded the MeVami-122 cassette, which is a miR-122 sequence flanked by 50 and 49 nucleotides of pri-miR-122 at its 5′ and 3′ ends, respectively. In this study, the miR-122 production was low when delivered by the OV, presumably because of the absence of Drosha translocation from nucleus to cytoplasm, which is required for the early stages of miRNA maturation. It is hypothesized that cytoplasmic miRNA precursors may mature through a currently unknown mechanism.

### 5.2. Some Other Findings on Oncolytic Viruses Encoding miRNAs

To our knowledge, there is only one study aimed at the investigation of an OV-encoding miRNA loaded with cytokine. The additive effect of miR-34a and interleukin IL-24 delivered by an oncolytic adenovirus was effective against HCC cells and led to the complete regression of tumors in mice [142].

In another study, the effect of OVs encoding artificial miRNAs was enhanced when combined with other drugs. In combination with GSK126, an EZH2 methyltransferase inhibitor, the oncolytic effect of VSVΔ51-amiR-4 against pancreatic tumors in mice was greater than that of either drug alone [38]. The effectiveness of the virus was also increased when combined with CTLA4 immune checkpoint inhibitors. An interesting effect was observed in this study: tumor cells not infected with OVs died as well, due to the transfer of amiR-4 into small extracellular vesicles from infected cells in vitro. It means that OVs expressing miRNAs can influence cells in the tumor microenvironment and kill cancer cells more effectively. Another OV, VSVΔ51-shPD-L1, which produced hairpin RNA targeting the immune response suppressor PD-L1, effectively suppressed tumor growth in syngeneic mice and did not exhibit toxic effects [38].

### 5.3. More Research Is Needed on OVs Expressing miRNAs

miRNAs for delivery by OVs are selected based on screens or literature data. Not all studies reveal the mechanism of microRNAs’ antitumor action delivered by OVs. However, some studies have shown that a number key genes were suppressed by miRNAs, leading to an anti-cancer effect. miRNAs inhibited genes associated with cancer development or led to the apoptosis of cancer cells; for example, the VV delivered miR-34a, which repressed the anti-apoptotic protein Bcl-2 and promoted the release of cytochrome c in myeloma cells [145]. The adenovirus similarly expressed miR-34a, which, in addition to Bcl-2, suppressed the SIRT1 oncogene [142], another adenovirus-encoded miR-143, which suppressed the expression of the KRAS gene, important in the progression of colorectal cancer [141]. Sometimes, in vitro efficacy studies of miRNA-expressing OVs differed from in vivo results [143,144].

## 6. Preclinical Studies of Oncolytic Viruses Expressing miRNA Decoys

### 6.1. Long Non-Coding RNAs as miRNA Inhibitors Delivered by Oncolytic Viruses

The number of free miRNAs in the cell is regulated by various molecules: pseudogenic RNAs, long non-coding RNAs (lncRNAs), circular RNAs (circRNAs), mRNAs, and viral transcripts [176]. Researchers use these or artificially created molecules to inhibit unwanted miRNAs in cells [177]. Clinical trials of miRNA inhibitors are currently underway for the treatment of genetic, infectious, oncological, and other diseases [61,178]. OVs are also used to deliver miRNA inhibitors to decrease the activity of oncogenic miRNAs in cells (Table 1). Viral systems are capable of not only effectively delivering inhibitors in vivo, but of also ensuring their long-term expression. To date, miRNA decoys were expressed mainly by adenoviruses in studies.

lncRNAs can act as inhibitors of miRNAs by complementarily binding to them [179], and can also compete with miRNAs for binding sites with target mRNAs, preventing translation inhibition [180]. lncRNA, synthesized in significant quantities by an OV, binds to a miRNA in cancer cells, thereby leading to a decrease in the number of free miRNAs and thus protecting target transcripts such as tumor suppressors. This approach enhances the antitumor activity of an OV.

An oncolytic adenovirus produced an artificial lncRNA containing sequences complementary to twelve different miRNAs associated with the development of HCC (miR21, miR221/222, miR224, miR17-5p/20a, miR10b, miR106b, miR151-5p, miR155, miR181a/181b, miR184, miR1, and miR501-5p) [149]. The adenovirus ensured the synthesis of a sufficient amount of lncRNA, which led to a change in the target miRNAs’ expression level in HCC cell lines and caused apoptosis. In HepG2 xenograft models, the OV resulted in a decreased tumor growth rate and the apoptosis of carcinoma cells.

In another communication [148], an oncolytic adenovirus encoded an artificial lncRNA with complementary sequences of nine miRNAs that regulate epithelial–mesenchymal transition (EMT) in triple negative breast cancer (TNBC) (mir-9-5p, miR10b- 5p, miR-21–5p, miR-23a-3p, miR-29a-3p, miR-155-5p, miR-222–3p, miR-301a-3p, and miR-373-3p). The proliferation of cancer cells expressing survivin was significantly reduced upon OV infection, and tumor size decreased in xenograft models of TNBC, while the expression pattern of EMT markers changed and the levels of tumor suppressive factors increased.

### 6.2. A Protein as miRNA Decoy Delivered by Oncolytic Viruses

Another option for suppressing miRNAs in cells to increase an OV’s proliferation was used by Rauschhuber C. et al. [181]. The oncolytic adenovirus expressed the P-19 inhibitor protein derived from the tomato bushy stunt virus, which binds double-stranded RNAs that function as short interfering RNAs and prevents their interaction with the RISC [181]. P19 promoted the replication of the adenovirus and the oncolytic effect was observable on cancer cell lines of the liver, lung, cervix, intestine, and glioblastoma. Doerner J. et al. created selectively replicating OVs based on adenovirus serotypes 1, 2, 5, and 6 expressing P19 [146]. The oncolytic effect of the viruses was compared in lung, osteosarcoma, pancreatic, liver, and colon cancer cell lines. In most cell lines, viruses expressing P19 showed enhanced oncolytic activity. The OVs based on serotypes 1, 2, and 6 significantly slowed tumor progression in A549 xenograft models in vivo compared to H101, the first recombinant OV approved by a regulatory agency [8].

### 6.3. miRNA Decoys Can Enhance the Antitumor Effect of Oncolytic Viruses

An interesting approach is the elevation of an OV’s effectiveness by suppressing miRNAs which inhibit the OVs’ proliferation. Raimondi G. et al. found that the inhibition of miR-222, the expression level of which is increased in neoplasms of pancreatic ductal adenocarcinoma (PDAC), leads to the sensitization of tumors to oncolytic adenovirus [147]. Based on these data, the authors created the AdNuPAR-E-miR222-S with sponges for miR-222, which led to a decrease in the amount and inactivation of miR-222 in the cell. The virus suppressed growth and resulted in the reduction of PDAC tumors in xenograft mouse models with a single intravenous dose and did not cause serious toxic effects.

### 6.4. A Combination of miRNA-Based Modification Approaches Is Possible

In the literature, there is an example of the simultaneous use of two approaches to OV modification—the introduction of miRTs into the viral genome and the expression of a miRNA inhibitor. Moshiri F. et al. used a recombinant adenovirus targeted with miR-199, which is reduced in HCC cells and increased in normal liver cells, and a recombinant adeno-associated virus to express a molecular sponge targeting the oncogenic miR-221 [150]. Sponges effectively induced apoptosis in HCC cells in vitro.

## 7. Clinical Studies of Oncolytic Viruses with miRNA Recognition Sites

According to the ClinicalTrials.gov database [182], more than one hundred clinical trials of OVs are currently being conducted, including several studies of OVs modified with miRTs to improve their cancer specificity (Table 2). OVs have mainly been studied as monotherapy, but also in combination with other drugs.

Phase I/II trials of the oncolytic adenovirus AdVince (ELC-100), developed at Uppsala University in Sweden, started in 2016 and are run by Elicera Therapeutics AB in collaboration with Uppsala University (NCT02749331) [183]. The study evaluates the safety of multiple OV injections into the hepatic artery in patients with neuroendocrine carcinomas of gastrointestinal, bronchial, or pancreatic origin that have metastasized to the liver. The specificity of the virus to tumors was established by placing the *E1A* gene under the control of the human chromogranin A gene promoter, and *E1A* was targeted with miRTs to miRNA-122, which is actively synthesized in the liver. Also, to enhance the transduction of cancer cells with low CAR expression, the capsid contained a peptide that facilitates cell penetration—protein transduction domain (PTD) from the trans-transcription protein activator (Tat) of the human immunodeficiency virus-1 (HIV-1). In preclinical studies, AdVince selectively replicated and killed patient carcinoma cells ex vivo, while, at the same concentrations, it did not cause cytotoxicity in human hepatocytes and did not cause minor inflammatory reactions in human blood [184]. The clinical trial was planned to evaluate the safety and maximum tolerated dose of the drug, as well as antitumor efficacy, replication profile, and humoral and cytokine-mediated immune responses. The phase 1 study included four doses of the drug, with three patients per dose, and no side effects have been reported from AdVince to date. A full report on the AdVince study is expected once it is fully completed. Trials have also begun to be carried out in Germany (Tübingen) [185] with the support of the Victory NET Foundation, Genf, Schweiz [186].

Most miRNA-targeted OVs in clinical trials are based on HSVs. In oncolytic HSV type 1 ONCR-177, four genes are controlled by different miRNAs: miR-124-3p, miR-1-3p, and miR-143-3p in the 3′UTR of the ICP4 gene, miR-128-3p, miR-219a-5p and miR-122-5p in the 3′UTR of the ICP27 gene, miR-217-5p, miR-137-3p, and miR-126-3p in the 3′-UTR UL8 gene, and miR-128-3p, miR-204-5p, and miR-219a-5p in the 3′-UTR of the ICP34.5 gene. Mutations were also introduced into the UL37 and US12 (ICP47) genes encoding tegument proteins, to ensure viral replication only in tumors. The virus retains the γ34.5 gene, which allows it to resist the effects of the interferon response, and is armed with genes encoding immunostimulatory molecules-IL12, Fms-like tyrosine kinase ligand 3 (FLT3L, extracellular domain), and C-C motif chemokine ligand 4 (CCL4), as well as control immune checkpoint inhibitors, namely anti-programmed cell death protein-1 nanoantibody (anti-PD-1) and anti-cytotoxic T-lymphocyte-associated protein 4 antibody (anti-CTLA-4, ipilimumab).

In preclinical studies, the insertion of miRTs resulted in active viral suppression comparable to the effect of acyclovir [187]. ONCR-177 exhibited significant oncolytic activity against human melanoma lines (A375, SK-MEL-28), colorectal carcinoma (SW-837, COLO 205), and SCCHN (FaDu, SCC25), and also against lysed lung carcinoma (H1299) and non-small-cell lung cancer (H1975) cell lines in the presence of interferon-alpha. At the same time, viral replication was suppressed in neurons, cardiomyocytes, and hepatocytes derived from pluripotent stem cells. When administered intratumorally in FaDu xenograft models, the virus actively replicated in tumors predominantly without spreading beyond the tumors. An identical OV with murine homologues of IL12, CCL4, and extracellular domain of FLT3LG, resulted in the complete or partial reduction of tumors in syngeneic bilateral mouse models, prolonged the life of mice, and induced the development of immune memory against cancer antigens.

ONCR-177 has been undergoing open-label, multicenter Phase 1 clinical trials conducted by Oncorus Inc. (Andover, MA, USA) since 2020, involving 66 patients (NCT04348916). ONCR-177 was tested in advanced and/or refractory cutaneous, subcutaneous, or metastatic nodal solid tumors or liver metastases from solid tumors as monotherapy and in combination with the PD1 inhibitor pembrolizumab. The purpose of the study was to evaluate the safety and tolerability of ONCR-177, determine the recommended dose of the drug for phase 2 studies, and determine the immune response. As a result, ONCR-177 was well-tolerated by patients, side effects were reported in 66.7% of patients and were the same as expected from viral therapy (inflammatory symptoms including chills, nausea, hypotension, and cytokine release syndrome), and no dose-limiting toxicity was observed [188]. Recommended doses for phase 2 studies of ONCR-177 as monotherapy and in combination with pembrolizumab were determined to be 4 × 10^8^ PFU in 4 mL once every 2 weeks (Q2W) for up to 26 doses.

The OV stimulated an antitumor immune response in the tumor microenvironment—patients exhibited a mild dose-dependent cytokine release syndrome, increased levels of IFNα, and the proliferation of T-lymphocytes in the blood, as well as the infiltration of immune cells into the tumor and tumor PD-L1 expression. Patients with triple-negative breast cancer had long-term disease stability, whereas patients with SCCHN and melanoma had early responses to therapy. No subjects discontinued treatment due to adverse events, and all treatment-related adverse events in combination with pembrolizumab were classified as grade 1–2.

Despite the positive results, further trials were canceled due to changes in the interests of the Oncorus Inc. [189].

Another OV, VG2025, is undergoing two phase 1 clinical trials at once, starting in 2022. It is a type 1 HSV that expresses the cytokines IL-12 and IL-15 and the alpha subunit of the IL-15 receptor under the control of the tumor-specific C-X-C chemokine receptor type 4 (CxCR4) promoter. Glycoprotein B (gB) was truncated to enhance cell-to-cell fusion, which helps the virus spread to target cancer cells. The native promoter of the ICP27 gene was replaced by the promoter of carcinoembryonic antigen (CEA), and the ICP34.5 gene was targeted by miR-124 and -143, which are actively synthesized in normal brain tissues and whose activity is reduced in some cancer tumors.

In preclinical studies, VG2025 led to a significantly higher expression of cytokines in A549 lung adenocarcinoma cells, compared to non-cancerous MRC-5 fibroblasts [134]. When injected into a mouse brain, the spread of the virus from the injection site was greatly suppressed. Although all xenograft models treated with VG2025 had at least a partial response, two mice in each of the two highest dose groups (1 × 10^5^ PFU and 1 × 10^6^ PFU) had tumors that reduced to undetectable levels by day 45. The murine analog of VG2025 and VG2026 caused complete tumor elimination in four of ten mice injected with A20 B-cell lymphoma cells on day 75 after the start of the treatment, while the significant activation of antitumor immunity was observed. The virus eliminated metastases when administered systemically, and the insertion of miRTs into the 3′UTR of the ICP34.5 gene prevented viral latent infection. Cytokines encoded by the virus virtually did not spread beyond the tumor. In single-dose acute toxicity studies in rhesus monkeys, all animals survived and there was no evidence of drug toxicity. The maximum tolerated dose of VG2025 in rhesus monkeys is equal to or greater than 1 × 10^9^ PFU/kg when administered subcutaneously and equal to or greater than 2 × 10^9^ PFU/kg when administered intravenously.

Clinical trials of VG2025 are in the patient recruitment phase. One is being conducted in China by Virogin Biotech Shanghai Ltd. (Virogin Biotech, Richmond, BC, Canada; NCT05477849) to evaluate VG2025 as a monotherapy against advanced solid tumors. Patients with colorectal cancer, duodenal cancer, hepatocellular carcinoma, pancreatic cancer, non-small cell lung cancer, intrahepatic cholangiocarcinoma, neuroendocrine carcinoma (NEC), and neuroendocrine cancer are being recruited.

As a result, no dose-limiting toxicity was observed, and there were no side effects that required dose reduction or led to the discontinuation of treatment, with fever being the most common [190]. There was a decrease in tumors outside the localized treatment—an abscopal effect. In patients with positive response, the expression of integrin signaling pathway genes, antigen-presenting MHC (HLA-DQA1 and HLA-DRB1) and chemokine receptor (CMKLR1) genes increased, as did the activation of CD8-positive T cells.

The second phase 1 clinical trial of the VG2025 virus is being conducted in Canada (Virogin Biotech Canada Ltd.) and the USA (MD Anderson Cancer Center, Houston, Texas, USA), where the effect of the OV in monotherapy and in combination with nivolumab on advanced solid tumors is being studied (NCT05266612). The study involves patients with duodenal adenocarcinoma, pancreatic cancer, ovarian cancer, leiomyosarcoma, salivary gland cancer, hepatocellular cancer, intrahepatic cholangiocarcinoma, colorectal cancer, and colon cancer [191]. VG2025 did not cause serious adverse events, the most common of which was fever, and no dose-limiting toxicity was observed. In patients, the expression of genes involved in the immune response changed and immune cells were activated.

## 8. Summary of the Current State of the Research

The combination of the miRNA interference mechanism and OVs is a novel and promising strategy to combat cancer. Three main strategies are being increasingly explored in the field of oncolytic virotherapy: (1) equipping oncolytic viruses (OVs) with recognition sites for miRNAs, (2) using OVs to deliver miRNAs, and (3) employing OVs to deliver miRNA inhibitors. Currently, the first approach is the most common, compared to the other two. However, several challenges remain regarding these methods. Despite a significant number of successful studies reporting miRNA-targeted OVs, the degree of OV inhibition in normal tissues was not always sufficient. miRTs insertions have the potential to destabilize viral genomes. Therefore, it is crucial to investigate miRT integrity and develop novel strategies to address this issue, even considering existing approaches. It remains unclear which specific miRNAs or their combination are most effective at inhibiting an OV within normal tissues, and how the number and placement of miRTs within the viral genome impact this inhibition while preserving the virus’s ability to kill cancer cells. However, OVs with miRTs are currently undergoing their first clinical trials, and their anticancer effect in combination with other drugs is being studied.

Variations in miRNA expression level in cancer cells using OV-encoded miRNA precursors or miRNA inhibitors is a promising new approach that has not yet reached the clinic. These modifications enhance the OVs’ anticancer effect themselves. However, not all studies have identified the antitumor mechanism of delivered miRNAs or miRNA inhibitors. To advance these agents from research to clinical practice, additional research is needed on their effects on the body and their safety.

To our knowledge, there are a few studies devoted to the comparison of miRNA-based modifications of OVs and other approaches to increase the effectiveness and safety of OVs. In one study [30], two approaches were compared on oncolytic mengovirus—the insertion of miRNA recognition sites and the deletion of the *leader* gene, which inhibits the activation of the type I IFN response pathway. Targeting the virus with miRNA target sites was significantly more effective than deleting the *leader* gene, in vivo. Another study compared the activity of vaccinia virus with a deleted thymidine kinase (TK) encoding gene, and a virus combining this approach with miRNA-detargeting [37]. Both viruses showed significant antitumor effects, although the effect of the double-regulated virus was slightly less. However, double-regulation alleviated viral toxicity much more than only TK-deletion, and led to a significantly higher increase in the life extension of tumor-bearing mice.

There are several studies that have combined the introduction of miRTs and other methods to improve OVs’ tumor selectivity [24,32,33,110,127,130,134,136].

One study combined the viral expression of tumor-suppressive miRNA-34 and IL-24 to improve an OV’s efficacy, and also compared the use of these approaches separately [142]. As a result, each approach had a similar antitumor effect, and their combination led to an enhanced antitumor response.

There are four clinical studies on OVs with miRTs, and three of them are at the stage of recruiting patients. This once again suggests that the method of increasing the tumor specificity of an OV through the RNA interference mechanism is effective and relevant.

## 9. Conclusions

miRNA-based modification methods have been shown to be effective in increasing the tumor selectivity and antitumor efficacy of OVs. In further studies, there will be more understanding of the optimal design of sequences containing miRNA target sites. Undoubtedly, bioinformatics research will play a significant role. It is also likely that complex genetic circuits for more precise virus control will be developed, containing targets for different miRNAs, as was demonstrated in one of the studies reviewed.

It is known that viruses and cells influence each other using miRNAs. Research findings on this interaction will be used to improve the effectiveness of OVs. A thorough understanding of gene expression alteration by OVs expressing miRNAs or miRNA decoys could help predict their far-reaching effects, since miRNAs delivered to cancer cells can also affect surrounding ones through some cell-to-cell communication mechanisms. In future studies, it is likely that OVs expressing several different miRNAs will emerge. As shown in one study, it is possible to combine miRNA expression or miRNA inhibitor approaches with miRTs.

Thus, the further investigation of OVs modified with miRNA-based methods will facilitate the creation of safer and more effective anti-cancer drugs. The combined approach of OV- and miRNA-based cancer therapy is at the beginning of its journey and will find its application in medicine.

## Figures and Tables

**Table 1:** Overview of oncolytic viruses, with miRNA-based modifications.

Virus	Viral Family	Oncolytic Virus	Genome	Target Cancer Type	Type of miR-Associated Modification	miR	Features	Combo Agent 1	Combo Agent 2	Reference
Coxsackievirus B3	Picornaviridae	PD-SK	Linear ss(+)RNA ^1^	Colorectal cancer	Target for miR	miRT-375 in the 3′ UTR of the genome	-	-	-	[132]
Coxsackievirus B3	Picornaviridae	miR-CVB3	Linear ss(+)RNA	Breast cancer and melanoma	Target for miR	Four copies of miRT-145, two copies of miRT-143, two copies of miR-1, and four copies of miR-216 in the 5′UTR of the CVB3 genome	-	Pore-forming lytic peptide melittin	Synthetic Toll-like receptor 9 ligand-CpG oligodeoxynucleotides	[123]
Coxsackievirus B3	Picornaviridae	miR-CVB3-1.1	Linear ss(+)RNA	Breast cancer and small-cell lung cancer	Target for miR	Four copies of miRT-145, two copies of miRT-143, two copies of miR-1, and four copies of miR-216 in the 5′UTR of the CVB3 genome	-	-	-	[124]
Coxsackievirus B3	Picornaviridae	PD-H-375TS	Linear ss(+)RNA	Colorectal cancer	Target for miR	Two copies of miRT-375 in the 3′UTR of the OV’s genome	-	-	-	[119]
Coxsackievirus B3	Picornaviridae	H3N-375TS with miRT-375 and H3N-375/1TS with miRT-375 and miRT-1	Linear ss(+)RNA	Colorectal carcinoma	Target for miR	miRT-375 and miRT-1	-	-	-	[120]
Coxsackievirus B3	Picornaviridae	CVB3-HP	Linear ss(+)RNA	Triple-negative breast cancer (TNBC)	Target for miR	miRT-1 and miRT-217 in the 3′UTR of the viral genome	-	-	-	[118]
Coxsackievirus B3	Picornaviridae	Sveveral viruses	Linear ss(+)RNA	Lung adenocarcinoma	Target for miR	Four copies of miRT-145 and two copies of miRT-143 in either the 5′ UTR or 3′ UTR of the CVB3 genome	-	-	-	[122]
Coxsackievirus A21	Picornaviridae	Several viruses	Linear ss(+)RNA	Melanoma	Target for miR	Two copies each of sequences completely complementary to miR-133 and miR-206, each separated by 4–6 nt long linkers in different positions of the viral genome	-	-	-	[125]
Coxsackievirus B3	Picornaviridae	Several viruses	Linear ss(+)RNA	Lung cancer	Target for miR	Four tandem miRT-34a and/or miRT-34c in the 5′ UTR or 3′ UTR of the CVB3 genome	-	-	-	[117]
Coxsackievirus B3	Picornaviridae	Several viruses	Linear ss(+)RNA	Non-specific	Target for miR	miRT-133 and miRT-206 in different positions of the viral genome	-	-	-	[121]
Adenovirus	Adenoviridae	Several viruses	Linear dsDNA ^2^	Hepatocellular carcinoma	Target for miR	miRT-21, miRT-199-3p, and miRT-142	GM-CSF, IL-2, and single-chain variable fragments (scFvs) against either programmed death-1 (PD-1) or programmed death-ligand 1 (PD-L1)	-	-	[34]
Adenovirus	Adenoviridae	Ad5-5miR145T and Ad5-10miR145T	Linear dsDNA	Breast cancer	Target for miR	Five or ten copies of miRT-145-5p downstream of *E1A* gene	GFP	-	-	[108]
Adenovirus	Adenoviridae	Conditionally replicative adenovirus (CRAd) l	Linear dsDNA	Hepatocellular carcinoma	Target for miR	miRNA-122 in 3′UTR of the *E1A* gene	Adenovirus loaded on human umbilical cord-derived mesenchymal stem cells, with *E1A* gene dual regulated by α-fetoprotein promoter and microRNA-122 target sequence.	-	-	[32]
Adenovirus	Adenoviridae	OAV-scIL12-miR, OAV-scIL12-GPI, and OAV-scIL12-TM	Linear dsDNA	Pancreatic cancer metastatic to the liver	Target for miR	miRT-122 in the 3′ UTR of the scIL-12 encoding sequence	scIL-12 encoding gene; OAV-scIL12-GPI contained GPI anchor signal from the folate receptor; OAV-scIL12-TM contained TM (transmembrane) domain from human CD4	-	-	[133]
Adenovirus	Adenoviridae	Ad-L5-8miR148aT	Linear dsDNA	Pancreatic cancer	Target for miR	Eight miRT-148a of perfect complementarity in the 3′UTR of the L5 coding sequence	-	-	-	[114]
Adenovirus	Adenoviridae	Several viruses	Linear dsDNA	Pancreatic ductal adenocarcinoma	Target for miR	miRT-148a and miRT-216a in the 3′UTR of the *E1A* gene	-	-	-	[107]
Adenovirus	Adenoviridae	A-4MREs	Linear dsDNA	Glioma	Target for miR	Two copies each of miRT-124, miRT-128, miRT-146b, and miRT-218 in the 3′UTR of the *E1A* gene	-	-	-	[111]
Herpes simplex virus	Herpesviridae	VG2025	Linear dsDNA	Non-specific	Target for miR	Tandem repeats of binding sites with perfect complementarity to miR-124 and miR-143 in 3′UTR of the ICP34.5 gene.	Truncated gB to enhance fusogenicity, control of ICP27 by CEA promoter; IL-12, IL-15, and the IL-15Rα subunit, CXCR4 promoter driving cytokine expression	-	-	[134]
Herpes simplex virus	Herpesviridae	ONCR-159	Linear dsDNA	Non-specific	Target for miR	miRT-128, miRT-204, and miRT-219a in 3′ UTR of the ICP34.5 gene; miRT-217, miRT-137, and miRT-126 in 3′ UTR of the UL8 gene; miRT-128, miRT-219a, and miRT-122 in 3′UTR of the ICP27 gene; and miRT-124, miRT-1, and miRT-143 in 3′UTR of the ICP4 gene	Deletion of the joint region, the introduction of a gateway recombination cassette in the UL3/UL4 intergenic region, a null mutation in US12, the mutation of amino acids D285N and A549T in the fusogenic glycoprotein B (gB), null mutations in ICP47, and mutations in UL37	-	-	[110]
Herpes simplex virus	Herpesviridae	3Ldn9T21	Linear dsDNA	Non-specific	Targets for miR: miR induces viral replication	miRT-21in UL9-C535C 3′UTR	-	-	-	[135]
Herpes simplex virus	Herpesviridae	SU4-124 HSV-1	Linear dsDNA	Glioma	Target for miR	Five copies of miRT-124 in 3′UTR of the ICP4 gene	ICP4 was placed under a tumor-specific survivin promoter and a 5′UTR of rat fibroblast growth factor 2 was added in front of the viral ICP4 gene	-	-	[33]
Herpes simplex virus	Herpesviridae	Several HSV-1 viruses	Linear dsDNA	Urothelial bladder cancer	Target for miR	Five copies each miRT-143 and/or miR-124 in 3′UTR of the ICP4 gene	-	-	-	[112]
Herpes simplex virus	Herpesviridae	KG4:T124, KGE-4:T124	Linear dsDNA	Glioblastoma multiforme	Target for miR	Four copies of miRT-124 in the 3′ UTR of ICP4 gene	Fusion of the complete gC ORF to GFP via a 2A peptide sequence; for KGE-4:T124, the introduction of scFv antibodies specific to EGFR	-	-	[136]
Semliki Forest virus	Togaviridae	SFV-AM6-124T	Linear ss(+)RNA	Glioblastoma	Target for miR	Six copies of miRT-124 between the viral nonstructural protein 3 and 4 (*NSP3* and *NSP4*) genes	4 aa substitutions as compared to the parental SFV4; that is, nsP3A70G, nsP3Y369F, nsP4R611K, and E2K162E	Anti-PD1 therapy	-	[24]
Semliki Forest virus	Togaviridae	SFV4B18RmiRT	Linear ss(+)RNA	Glioblastoma	Target for miR	Two copies of each miRT-124, miRT-125, and miRT-134 in the beginning of 3′UTR of the SFV genome	*B18R* gene coding for a decoy receptor, which binds to type-I IFN	-	-	[137]
Semliki Forest virus	Togaviridae	SFV4miRT	Linear ss(+)RNA	Glioblastoma multiforme and high-risk neuroblastoma	Target for miR	Two copies of each miRT-124, miRT-125, and miRT-134 in the beginning of 3′UTR of the SFV genome	-	-	-	[91]
Semliki Forest virus	Togaviridae	SFV4	Linear ss(+)RNA	Gliomas	Target for miR	miRT-124	-	-	-	[138]
Measles virus	Paramyxoviridae	MeV-CD-FmiRTS148a	Linear ss(-)RNA	Pancreatic ductal adenocarcinoma	Target for miR	miRT-148a in 3′UTR of the *fusion* (F) gene	Cytosine deaminase–uracil phosphoribosyl transferase	5-fluorocytosine (5-FC)	-	[139]
Measles virus	Paramyxoviridae	Several viruses	Linear ss(-)RNA	Non-specific	Target for miR	Viruses with target sites for miR-124-3p, miR-125b-5p, and miR-7-5p in the 3′UTRs of either the N, F, H, or L genes	EGFP	-	-	[109]
Measles virus	Paramyxoviridae	MV-EGFPmtd (multi-tissue-detargeted)	Linear ss(-)RNA	Pancreatic carcinoma	Target for miR	Three copies of fully complementary miRT-148a in 3′UTR of the F gene; three copies each of fully complementary miRT-122 and miRT-7 in 3′UTR of the hemagglutinin (H)	EGFP	-	-	[113]
Mengovirus	Picornaviridae	Several viruses	Linear ss(+)RNA	Myeloma, plasmacytoma	Target for miR	Two copies each of miRT-133b, miRT-208a, and miRT-124	Varying polyC tract lengths	-	-	[130]
Mengovirus	Picornaviridae	vMC24NC, vMC24ΔL	Linear ss(+)RNA	Glioblastoma	Target for miR	Two copies of miRT-124 in the 5′ UTR and two copies each of miRT-133 and miRT-208 in the 3′ UTR of the viral genome	vMC24ΔL-deletion of the *leader* gene, truncated polyC	-	-	[30]
Mengovirus	Picornaviridae	Several viruses	Linear ss(+)RNA	Non-specific	Target for miR	Several combinations of miRT-124, miRT-125, miRT-133, miRT-208, and miRT-142 in 3′UTR and/or 5′UTR	Truncated polyC	-	-	[127]
Enterovirus A71	Picornaviridae	EV-A71-miR124T	Linear ss(+)RNA	Malignant gliomas	Target for miR	Three copies of miR124T in the location between the 5ʹ-UTR and the coding sequences	-	-	-	[140]
Vaccinia virus	Poxviridae	ΔTK let-7a-targeted VV	Linear dsDNA	Multiple myeloma	Target for miR	miRT-let-7a in the 3′ UTR of *B5R* gene	Deleted thymidine-kinase	-	-	[37]
Adenovirus	Adenoviridae	Several viruses	Linear dsDNA	Non-specific	miR precursor	miR-1-3p in short hairpin RNA (shRNA), precursor microRNA (pre-miRNA), and primary miRNA (pri-miRNA) format; miR-26b-5p in pri-miRNA format	A 24 bp deletion in the early gene *E1A*	-	-	[87]
Adenovirus	Adenoviridae	ICOVIR15 miR-99b, ICOVIR15 miR-485	Linear dsDNA	Pancreatic cancer	miR precursor	pri-miR-99b and pri-miR-485—miRNA genomic sequences under the control of the cytomegalovirus promoter	A 24 bp deletion and E2F-responsive elements in the *E1A* region; the fiber contains an RGD motif inserted in the HI-loop region.	-	-	[85]
Adenovirus	Adenoviridae	Ad-ZD55-miR-143	Linear dsDNA	Colorectal cancer	miR precursor	miR-143 in pri-miRNA format	E1B55kDa encoding gene was deleted	-	-	[141]
Adenovirus	Adenoviridae	AdCN205-IL-24-miR-34a	Linear dsDNA	Hepatocellular carcinoma	miR precursor	miR-34a	*IL24* gene controlled by the adenovirus endogenous E3 promoter	-	-	[142]
VSV	Rhabdoviridae	rVSV-miR143	Linear ss(-)RNA	Osteosarcoma	miR precursor	miR-143	-	-	-	[143,144]
VSV	Rhabdoviridae	VSV∆51-amiR-4, VSVΔ51-shPD-L1	Linear ss(-)RNA	Non-specific	miR precursor	Artificial “amiR-4” or hairpin RNA targeting the immune response suppressor PD-L1	-	GSK126	-	[38]
Measles	Paramyxoviridae	Several viruses	Linear ss(-)RNA	Non-specific	miR precursor	Precursor miR-122 sequence flanked by 50 and 49 nucleotides of pri-miR-122 at its 5′ and 3′ ends, respectively.	-	-	-	[80]
Vaccinia virus	Poxviridae	VV-miR-34a	Linear dsDNA	Multiple myeloma	miR precursor	miR-34a in pri-miRNA format inserted into the thymidine kinase (TK) gene	Disrupted sequence of TK	VV encoding SMAC	-	[145]
Adenovirus	Adenoviridae	Several viruses	Linear dsDNA	Non-specific	miR inhibitors	Plant-derived RNAi inhibitor P19 under the control of the major late promoter and connected to the fiber coding sequence	A 24 bp deletion in the pRB binding region in the early gene *E1A*	-	-	[146]
Adenovirus	Adenoviridae	AdNuPAR-E-miR222-S	Linear dsDNA	Pancreatic cancer	miR inhibitors	miR-222 sponges	-	-	-	[147]
Adenovirus	Adenoviridae	AdSVP-lncRNAi9	Linear dsDNA	Triple negative breast cancer	miR inhibitors	Artificial lncRNA, which contains 10 copies of the complementary sequences of nine OncomiRs: mir-9-5p, miR10b-5p, miR-21–5p, miR-23a-3p miR-29a-3p, miR-155-5p, miR-222–3p, miR-301a-3p, and miR-373-3p	A 24 bp deletion in the early gene *E1A*	-	-	[148]
Adenovirus	Adenoviridae	AdSVPE1a-lncR	Linear dsDNA	Hepatocellular carcinoma	miR inhibitors	An artificially designed lncRNAi with a tandem of complementary binding sequences to the seed sequences of the 12 OncomiRs: miR21, miR221/222, miR224, miR17-5p/20a, miR10b, miR106b, miR151-5p, miR155, miR181a/181b, miR184, miR1, and miR501-5p. The sequences were repeated six times.	*E1A* gene under tumor-specific survivin promoter	-	-	[149]
Adenovirus and AAV ^3^	Adenovirus–Adenoviridae; AAV–Parvoviridae	rAd-199T-miR-221 sponge, rAAV.miR-221 sponge	Adenovirus–linear dsDNA; AAV–linear ssDNA	Hepatocellular carcinoma	miR inhibitors, target for miR	mir-221 sponge with four copies of the miR-221 binding sites and four-nucleotide-long spacers; the adenivirus contained miRT-199	-	-	-	[150]

^1^ ss—single-stranded; ^2^ ds—double-stranded; ^3^ AAV—adeno-associated virus.

**Table 2:** Overview of clinical trials of microRNA-detargeted oncolytic viruses.

Virus	Oncolytic Virus Name	miRTs	Features	Clinical Trial (ID)	Monotherapy or Combination	Combo Agent	Cancer Type	Phase	Recruitment Status
Adenovirus	AdVince	miRT-122 in 3′UTR of the *E1A* gene	Gene promoter from human chromogranin A, a cell-penetrating peptide in the capsid (the protein transduction domain (PTD) from the trans-activator of transcription (Tat) protein of human immunodeficiency virus (HIV)-1)	NCT02749331	Mono	No	Metastatic neuroendocrine tumors (NETs)	1/2	Recruiting
HSV	ONCR-177	miRT-124-3p, miR-T-1-3p, and miR-T-143-3p in 3′UTR of the ICP4 gene; miR-T-128-3p, miR-T-219a-5p, and miR-T-122-5p in 3′UTR of the ICP27 gene; miR-T-217-5p, miR-T-137-3p, and miR-T-126-3p in 3′UTR of the UL8 gene; and miR-T-128-3p, miR-T-204-5p, and miR-T-219a-5p in 3′UTR of the ICP34.5 gene	Genes coding for IL12, FLT3LG, CCL4, PD-1, CTLA-4, and UL37 mutation	NCT04348916	Combo	Pembrolizumab	Melanoma, solid tumors, head and neck squmous cell carcinoma, and breast cancer	1	Terminated
HSV	VG2025	miRT-124 and miRT-143 in 3′UTR of the ICO34.5 gene	CXCR4 promoter driving cytokine expression, truncated gB to enhance fusogenicity, control of ICP27 gene by CEA promoter; IL-12, IL-15, and the IL-15Rα subunit coding sequences	NCT05477849	Mono	No	Advanced malignant solid tumor	1	Recruiting
HSV	VG2025	miRT-124 and miRT-143 in 3′UTR of the ICO34.5 gene	CXCR4 promoter driving cytokine expression, truncated gB to enhance fusogenicity, control of ICP27 gene by CEA promoter; IL-12, IL-15, and the IL-15Rα subunit coding sequences	NCT05266612	Mono, combo	Nivolumab	Advanced malignant solid tumor	1	Recruiting

## Data Availability

Not applicable.

## Supplementary Materials

The following supporting information can be downloaded at: https://www.mdpi.com/article/10.3390/pharmaceutics16080986/s1, Table S1: “MicroRNAs selected for modification of oncolytic viruses” [192,193,194,195,196,197,198,199,200,201,202,203,204,205,206,207,208,209,210,211,212,213,214,215,216,217,218,219,220,221,222,223,224,225,226,227,228,229,230,231,232,233,234,235,236,237,238,239,240,241,242,243,244,245,246,247,248,249,250,251,252,253,254,255,256,257,258,259,260,261,262,263,264].
